# Iniciativa educativa con adolescentes de México y conducta suicida

**DOI:** 10.15446/rsap.V25n3.104060

**Published:** 2023-05-01

**Authors:** Nancy L. González-Cruz, Luz Arenas-Monreal, Elisa Hidalgo-Solórzano, David Hernández-Bonilla, Claudia M. Vega-González, Amanda Bravo-Hernández, Rosario Valdez-Santiago

**Affiliations:** 1 NG: Lic. Enfermería. M. Sc. Salud Pública. Escuela de Salud Pública de México/Instituto Nacional de Salud Pública. Cuernavaca, México. nancyglz_14@hotmail.com Instituto Nacional de Salud Pública Escuela de Salud Pública de México Instituto Nacional de Salud Pública Cuernavaca Mexico; 2 LA: Lic. Medicina. Ph. D. Antropología Médica. Instituto Nacional de Salud Pública. Cuernavaca, México. luz.arenas@insp.mx Instituto Nacional de Salud Pública Antropología Médica Instituto Nacional de Salud Pública Cuernavaca Mexico luz.arenas@insp.mx; 3 EH: Lic. Medicina. M. Sc. Ciencias. Instituto Nacional de Salud Pública. Cuernavaca, México. elisa.hidalgo@insp.mx Instituto Nacional de Salud Pública Instituto Nacional de Salud Pública Cuernavaca Mexico elisa.hidalgo@insp.mx; 4 DH: Lic. Psicología Clínica Infantil. M.Sc. Neuropsicología Clínica Infantil. M.Sc. Salud Pública. Ph.D. Epidemiología. Instituto Nacional de Salud Pública. Cuernavaca, México. david.hernandez@insp.mx Instituto Nacional de Salud Pública Instituto Nacional de Salud Pública Cuernavaca Mexico david.hernandez@insp.mx; 5 CV: Lic. Psicología y Pedagogía. M. Sc. Investigación Social Interdisciplinaria. Organización Fe y Alegría. Bogotá, Colombia. marcevega31@gmail.com Investigación Social Interdisciplinaria Organización Fe y Alegría Bogotá Colombia; 6 AB: Lic. Psicología. M.Sc. Neuropsicología y Educación. Consultora Independiente. Bogotá, Colombia. amybravo@gmail.com Neuropsicología y Educación Bogotá Colombia; 7 RV: Lic. Psicología. Ph. D. Ciencias en Salud Pública. Instituto Nacional de Salud Pública. Cuernavaca, México. rosario.valdez@insp.mx Instituto Nacional de Salud Pública Ciencias en Salud Pública Instituto Nacional de Salud Pública Cuernavaca Mexico

**Keywords:** Adolescentes, habilidades para la vida, conducta suicida *(fuente: DeCS, BIREME)*, Adolescents, life skills, suicidal behaviour *(source: MeSH, NLM)*

## Abstract

**Objetivo:**

Identificar la prevalencia de suicidio y llevar a cabo una iniciativa educativa con adolescentes de una escuela pública, a fin de fortalecer las habilidades para la vida (HpV) que les permitan enfrentar situaciones críticas de la vida cotidiana.

**Material y Método:**

Estudio realizado de septiembre del 2017 a julio del 2018, con enfoque cuantitativo y cualitativo e iniciativa educativa, con mediciones pre-postest de un solo grupo, en estudiantes de primer año de secundaria de una escuela pública ubicada en Morelos, México. Se aplicaron dos cuestionarios: 1) Cuestionario de indicadores psicosociales para depresión y riesgo suicida y 2) Cuestionario de HpV, además de grupos focales (GF) y registro etnográfico. Se efectuaron 12 sesiones educativas enfocadas en cuatro HpV.

**Resultados:**

Participaron 26 estudiantes. La prevalencia de ideación e intento de suicidio fue de 12,5% (IC 95% 3,5-28,9). Hubo significancia estadística en la habilidad de "Conocimiento de sí mismo", para hombres y mujeres. Además, en estas últimas también hubo dicha significancia en "Manejo de emociones" y "Puntuaciones globales". En los GF manifestaron que las HpV les estaban apoyando para su vida diaria.

**Conclusiones:**

Trabajar con adolescentes desde la propuesta de HpV les proporciona elementos para un mejor manejo de la vida diaria y puede contribuir a la prevención de la conducta suicida.

## Abstract (en)

**Objective:**

Identify the prevalence of suicide and carry out an educational initiative with adolescents from a public school to strengthen Life Skills (HPV) that allow them to face critical situations in daily life.

**Material and Method:**

Study conducted from September 2017 to July 2018, with a quantitative and qualitative approach and educational initiative with pre-posttest measurements of a single group, in first-year high school students from a public school located in Morelos. Two questionnaires were applied 1) Questionnaire of Psychosocial Indicators for Depression and Suicidal Risk and 2) Questionnaire of HpV, in addition to focus groups (FG) and ethnographic registry. Twelve educational sessions focused on four HPVs were held.

**Results:**

26 students participated. The prevalence of suicide ideation and attempt was 12.5% (95% CI 3.5-28.9). There was statistical significance in the ability "Self-knowledge" for men and women, in addition to the latter in "Managing emotions" and "Global Scores". In the FG it was established that the HPVs were supporting them in their daily life.

**Conclusions:**

Working with adolescents through the HpV proposal, provides them with elements for a better management of daily life and can contribute to the prevention of suicidal behavior.

El suicidio es un problema de salud pública. El intento de suicidio es uno de los componentes de la conducta suicida (CS) y se le considera el factor predictor más importante para el suicidio consumado 1. En un metaanálisis reciente se reportó una prevalencia de intento de suicidio en niños y adolescentes de 6% (IC 95 % 4,7-7,7%) 2. En México, el intento de suicidio en adolescentes ha incrementado, como lo muestran las prevalencias de las Encuestas Nacionales de Salud y Nutrición (Ensanut) del 2006 (1,1%), 2012 (2,7%) y 2018 (3,9%) 3.

El énfasis en el intento de suicidio permite enfocarse en la aplicación de programas de educación para la salud dirigidos a adolescentes para prevenir esta problemática; en este sentido, la educación para la salud es una de las herramientas metodológicas de la promoción de la salud a partir de la cual se fortalecen las capacidades de la población 4.

Existen diversas metodologías de educación para la salud, como por ejemplo el enfoque de Habilidades para la Vida (HpV), propuesto por la Organización Mundial de la Salud (OMS), que se basa en el aprendizaje de conocimientos, así como en la práctica y la aplicación de habilidades 5.

Una de las fortalezas del enfoque de HpV es que se fundamenta en diversas teorías del comportamiento, como la teoría social cognitiva (TSC), que parte del supuesto de que las conductas de las personas no están determinadas únicamente por factores intrínsecos, sino que son el resultado de la interacción con su ambiente 6. Este enfoque también se apoya en teorías del aprendizaje, como el constructivismo, que resaltan el papel activo de las personas en el proceso de enseñanza-aprendizaje en el contexto social 7.

En la adolescencia, la persona se desarrolla física, cognitiva y socialmente, sin embargo, este periodo conlleva riesgos que pueden comprometer la integridad, al grado que en México los accidentes, a pesar de que se han reducido paulatinamente, siguen ocupando las primeras causas de muerte en este grupo etario, y las lesiones intencionales han ido aumentando de manera significativa en la última década tanto en hombres como en mujeres 8. Por lo anterior, el desarrollo y fortalecimiento de las HpV cobra relevancia durante esta etapa.

Las iniciativas educativas con el enfoque de HpV en el ámbito escolar muestran mejoría en las aptitudes sociales y la convivencia y disminuyen las conductas agresivas. Asimismo, las iniciativas que incorporan el desarrollo de habilidades han mostrado ser más efectivas que las que se centran únicamente en la transferencia de información 9.

Se reportan diversos estudios en HpV, de los cuales algunos se enfocan en la identificación y la validación de escalas de HpV 10-11, en tanto que otros se basan en iniciativas educativas para fortalecer las HpV en población infantil y adolescente 12-19. Otros estudios utilizan este enfoque con la finalidad de prevenir una problemática específica, como lo son las adicciones 20-21 y el embarazo adolescente 22, así como para mejorar la salud mental, evitar el estrés, el *bullying* y el suicidio 23-26.

El objetivo de este estudio fue identificar la prevalencia de suicidio e implementar una iniciativa educativa con adolescentes de una escuela pública en México, a fin de fortalecer HpV que les permitan hacer frente a situaciones críticas de la vida cotidiana.

## MATERIAL Y MÉTODO

Estudio transversal que utilizó un enfoque cuantitativo y cualitativo y una iniciativa educativa con mediciones pre-pos test de un solo grupo, en estudiantes de primer año de secundaria de una escuela pública en un municipio del estado de Morelos. Se realizó de septiembre del 2017 a julio del 2018. Este estudio hace parte de una investigación más amplia enfocada en la CS de adolescentes en México, realizada en cinco entidades federativas del país.

### Instrumentos y técnicas de recolección de información

1. Cuestionario de Indicadores Psicosociales para Depresión y Riesgo Suicida (CIP-DERS), constituido por 86 preguntas en seis secciones: 1) datos parasuicidas, 2) ideación suicida, 3) relaciones con mamá/papá, 4) sintomatología depresiva, 5) impulsividad y 6) consumo de sustancias 27.

2. Cuestionario de HpV, conformado por 43 preguntas con cinco secciones: 1) datos sociodemográficos, 2) conocimiento de sí mismo, 3) comunicación efectiva, 4) empatía y 5) manejo de emociones 28.

Se hizo registro etnográfico y un grupo focal (GF) pre/ post iniciativa, conformado por ocho escolares de ambos sexos, y se diseñaron guías que incluyeron seis ejes temáticos relacionados con las interacciones sociales en el ámbito escolar y las HpV. La selección de quienes participaron en los GF se basó en que fueran estudiantes con facilidad para socializar y entablar conversación.

### Etapas de la iniciativa

#### Planeación

La directiva de la escuela seleccionó uno de los cuatro grupos de primer año, en quienes consideraban necesario fortalecer HpV Se aplicaron los cuestionarios CIP-DERS, HpV y se realizó el GF pre.

Con la información obtenida del GF se seleccionaron cuatro habilidades para desarrollarse en 12 sesiones: conocimiento de sí mismo, empatía, manejo de emociones y comunicación efectiva. El diseño de las sesiones se fundamentó en los constructos de aprendizaje vicario y autoeficacia de la TSC y en los fundamentos teóricos del constructivismo 6-7.

#### Implementación

Se desarrollaron 12 sesiones de 50 min cada una, con metodología participativa (Cuadro 1), las cuales se llevaron a cabo en las instalaciones de la escuela, de enero a abril del 2018. Las sesiones fueron coordinadas por una pareja de facilitadoras educativas (integrantes del equipo de investi gación). Durante las sesiones se hizo registro etnográfico.

**Cuadro 1:** Sesiones educativas, habilidades para la vida y actividades

Sesión	Habilidad	Nombre Actividad	Actividades
1	Conocimiento de sí mismo	¿Quién soy?	Se formaron parejas; a una persona de cada pareja se le vendaron los ojos, mientras las otras personas cambiaban lugares y preguntaban a su nueva pareja: ¿Quién soy yo? (podían fingir o cambiar el tono de voz), la persona con los ojos vendados debía adivinar quién era su compañera/o en turno. Al cabo de 5 seg, se movían con otra/o compañera/o al azar. Posteriormente, se invirtieron los roles y se repitió la secuencia. Finalmente, se reflexionó y retroalimentó sobre la dinámica y habilidad practicada.
2		Ficha de identidad	Se entregó una "ficha de identidad" que el alumnado llenó con datos como: hobbies, gustos, disgustos, habilidades, fortalezas y áreas de oportunidad. Finalmente, se reflexionó y retroalimentó sobre la dinámica y habilidad practicada.
3		Adivina mi perfil	El alumnado llenó un formato estilo Facebook, con: cualidades físicas, habilidades, cosas que les gustaba mucho, pero sin poner su nombre. Los perfiles fueron repartidos al azar al alumnado, cada uno leyó el perfil y anotó el nombre de la persona a la que creía que pertenecía. En seguida, algunas personas leyeron en voz alta el perfil y el nombre de la persona a la que creía que pertenecía y sus razones, el otro alumno/a confirmaba, o no, si el perfil era suyo. Posteriormente, el resto del grupo se paró a entregar el perfil a la persona que creía que correspondía. Finalmente, se reflexionó y retroalimentó sobre la dinámica y habilidad practicada.
4	Empatía	Armando la historia	Se pegaron cuatro rotafolios con los siguientes encabezados: "¿Quién y cómo es (nombre facilitadora)?"; "Lo que hizo la tarde anterior fue..."; "Lo que hizo la noche anterior fue..."; "Lo que hizo esa mañana fue...". Debajo, el rotafolio estaba dividido en dos partes: A) Lo que creo que es/pasó y B) Lo que es/pasó. Se formaron cuatro equipos y se les pidió que "armaran" la historia de lo que había pasado la facilitadora antes de su llegada al salón de clases en la primera sesión, escribiendo en los post-it lo que creían según el encabezado del rotafolio asignado y los pegaron en el lado "A". Posteriormente, la facilitadora leyó en voz alta lo del lado "A" a manera de narración, y en el lado "B", fue escribiendo lo que en realidad era o pasó en cada situación. Finalmente, se reflexionó y retroalimentó sobre la dinámica y habilidad practicada.
5		Preguntas importantes	Se les pidió que, de manera individual, escribieran 10 preguntas que consideraran importantes para conocer a las/os demás, abarcando todas las esferas vitales (familia, sueños-ilusiones, amigos, sexualidad, creencias, situaciones difíciles, situaciones bonitas, etc). Una vez terminado el listado de preguntas, se explicó la técnica DRER: Detenerse, Respirar, Escuchar y Responder (¿Cómo te puedo ayudar? ¿Qué quieres hacer?). Se pidió que formaran parejas y se hicieran esas preguntas el uno al otro, utilizando la técnica DRER pero sólo los primeros tres pasos, en el cuarto paso "responder" se le agradeció al compañero/a por compartir con ellos. Se recalcó en todo momento la importancia de escuchar con respeto al compañero/a, sin burlas. Finalmente, se reflexionó y retroalimentó sobre la dinámica y habilidad practicada.
6		Reconocimiento a la empatía	Se entregó a cada alumna/o dos stickers y se les pidió que lo entregaran a dos compañero/as que mostraba más empatía con ellas/os y/o con los demás. Se felicitó con un aplauso a quienes recibieron más stickers, y se les preguntó si sabían que estaban siendo empáticos, también se preguntó las razones por las que entregaron los stickers. Se invitó a seguir el ejemplo de quienes que con algunas acciones hicieron sentir bien a los demás.
		Y si yo fuera...	Se formaron nueve equipos de cuatro alumnos y se les asignó aleatoriamente dos situaciones ficticias de jóvenes en diferentes circunstancias, en los que desarrollaron las siguientes preguntas: ¿Cómo me sentiría yo si estuviera pasando por eso?, ¿Cómo me gustaría que los demás fueran conmigo si yo estuviera pasando por eso?, ¿Cómo me gustaría que mis amistades me apoyaran si yo estuviera pasando por eso? Un equipo expuso de manera verbal al resto del grupo el caso y las respuestas a las tres preguntas. Finalmente, se reflexionó y retroalimentó sobre la dinámica y habilidad practicada.
		Reconociendo emociones	Se dividió al grupo en dos y en el centro del salón se colocó un timbre. En cada turno pasaba una persona de cada equipo y se les mostraron diferentes imágenes, primero de escenas con personas, luego de rostros completos, finalmente de partes del rostro, y en cada imagen se pidió que identificara la emoción expresada. Ganó el equipo que tuvo mayores aciertos. Finalmente, se reflexionó y retroalimentó sobre la dinámica y habilidad practicada.
7	Manejo de emociones	Pasa la emoción	Se acomodaron en círculo. Se hicieron tarjetas con secuencias de emociones (ejemplo: tristeza-alegría-alegría-rabia-desagrado) únicamente quién iniciaba la actividad podía leer la tarjeta, después sólo tenía que gesticular la secuencia de las emociones a la persona de al lado y así hasta pasar por todas/os los compañeros. El último tuvo que gesticular y nombrar las emociones según la secuencia y se comparó si fue igual a la inicial Finalmente, se reflexionó y retroalimentó sobre la dinámica y habilidad practicada.
8		Expresiones	Se formó un grupo de hombres y otro de mujeres. Se le entregaron dos rotafolios a cada equipo y dibujaron en cada uno a un hombre y una mujer. Escribieron las emociones vistas en sesiones pasadas y cómo lo expresan en cada caso. Cada equipo expuso y el otro equipo comentó si estaba de acuerdo con lo expuesto. Finalmente, se reflexionó y retroalimentó sobre la dinámica y habilidad practicada.
9		Negativo a positivo	Se formaron cinco equipos y a cada uno se le asignó una emoción. Se pidió que dibujaran una situación de la vida cotidiana en la que pudieran experimentar esa emoción y pensaran en la respuesta a las siguientes preguntas: ¿cómo lo siento? ¿Cómo reacciona mi cuerpo? ¿Cómo esa emoción se expresaría de manera negativa? al expresarlo así, ¿puedo herir los sentimientos de alguien más, o hacerlos sentir mal? ¿Cómo esa emoción se expresaría de manera positiva? Cada equipo presentó al resto del grupo su dibujo y sus respuestas. Finalmente, se reflexionó y retroalimentó sobre la dinámica y habilidad practicada.
10	Comunicación efectiva	Rally de comunicación	Se formaron tres equipos, cada equipo se sentó en filas paralelas. A la persona del final de cada fila se le dio una tarjeta con un dibujo o palabra, y esta a su vez debía pasar el mensaje, es decir, escribió o dibujó en la espalda del de enfrente con la ayuda de sus dedos, y así se fueron pasando el mensaje hasta llegar al inicio de la fila, donde debían escribir o dibujar el mensaje recibido. Se repitió cambiando posiciones y mensajes. Posteriormente, los mismos equipos se acomodaron en círculo. A quien inicie, se le dio una tarjeta con un mensaje que deberá pasar, pero con mímica, y la última persona escribió lo que creía que le quisieron decir. Esto se repitió con un mensaje distinto. Por último, se les dio otra tarjeta a otra persona y esta debía recordar el mensaje textual y pasarlo, esta vez solo hablando, la última persona debía escribir el mensaje que le dijeron. Esto se repitió con un mensaje distinto. Ganó el equipo con mayor cantidad de mensajes acertados. Finalmente, se reflexionó y retroalimentó sobre la dinámica y habilidad practicada.
11			Se formaron tres equipos y se les asignó un caso ficticio y un rol: asertivo, pasivo o agresivo; en función del Las tres formas rol, formularon su respuesta al caso y lo dramatizaron al resto del grupo. Finalmente, se reflexionó y retroalimentó sobre la dinámica y habilidad practicada.
12		Consigue el papel	Formaron parejas y a una persona de cada pareja se les pidió salir un momento del salón. A quienes quedaron adentro, se les pidió que en un papel escribieran las tres cosas o personas más importantes en su vida y que pisaran el papel. Se pidió que cuidaran ese papel y no se lo entregaran a su compañero/a a menos que, con argumentos o dialogando, les convencieran (por ejemplo, pedir por favor). A quienes estaban afuera se les dijo que su compañero/a tenía algo debajo de sus pies y que debían conseguirlo a como diera lugar, tenían 1 minuto para lograrlo, la única regla fue no agredir físicamente. Pasado el tiempo, se pidió que tomaran asiento y se les explicó cómo pudieron haber obtenido el papel fácilmente. Finalmente, se reflexionó y retroalimentó sobre la dinámica y habilidad practicada.
		Comunicar positivismo	Como actividad final del curso, se le entregó a cada persona unos post it, en donde escribieron algún mensaje positivo a quienes quieran del salón, una vez terminado de escribir, fueron con cada uno de sus compañeros/as a quienes les hayan escrito, les pegaron el postit, les leyeron el mensaje y se abrazaron si así lo deseaban. Se agradeció la participación en la sesión y en todo el curso.

#### Evaluación

Un mes después de finalizadas las sesiones, se aplicó el cuestionario de HpV y se realizó otro GF con las mismas personas del GF anterior.

#### Análisis

Cuestionario CIP-DERS. Se utilizó el programa STATA 14.1®, se realizó una descripción general de las variables: distribución, presencia de valores fuera de rango y valores perdidos. Se obtuvo la proporción por categoría (prevalencia de intento e ideación suicida). Se estimaron medidas de tendencia central y de dispersión para variables cuantitativas discretas (edad, edad del primer intento, promedio de intentos).

Las puntuaciones de las diferentes dimensiones del cuestionario de HpV fueron analizadas comparando el pre y el pos test para mujeres y hombres mediante la prueba Wilcoxon; se utilizó el programa STATA 14.1®.

Los GF fueron audiograbados y posteriormente transcritos, al igual que el registro etnográfico; se identificaron códigos y categorías. Se utilizó el software Atlas ti v8 para la codificación y posteriormente se crearon matrices para identificar regularidades y diferencias.

#### Consideraciones éticas

Esta iniciativa forma parte del proyecto "Conducta suicida en adolescentes en México", coordinado por investigadoras del Instituto Nacional de Salud Pública (INSP), el cual contó con financiamiento del Consejo Nacional de Ciencia y Tecnología (proyecto 273115). Fue autorizado por el Comité de Ética en Investigación del INSP; se contó con la autorización de los directivos de la escuela, con el consentimiento de madres/padres de familia y con el asentimiento de los estudiantes.

## RESULTADOS

### Características de los estudiantes

Participaron 26 estudiantes que respondieron el cuestionario pre y pos, 14 mujeres y 12 hombres. El promedio de edad fue de 12 años, el 57,6 % vivía con ambos padres, el promedio de integrantes de la familia fue de 5,6 integrantes. El 45,5 % reportó que algunas veces había discusiones en la familia por falta de dinero.

### Conducta suicida

La prevalencia de la ideación y del intento de suicidio fue de 12,5% (IC 95% 3,5-28,9). La edad promedio de quienes reportaron haberse lastimado con el fin de quitarse la vida fue de 11,2 años. Para la ideación suicida, el 13,3% eran mujeres y para el intento suicida el 17,7% eran hombres. Este era el primer intento de quitarse la vida. El 25% respondieron haber utilizado un objeto punzocortante, el 25% se provocó trauma y el 50% no respondió.

### Conocimiento de sí mismo

Los participantes del GF reportaron que hubo un cambio posterior a la iniciativa, que se reflejó en la forma en la que manifiestan y manejan las HpV (Cuadro 2).

**Cuadro 2:** Sesiones educativas, habilidades para la vida y objetivos de cada sesión

Sesión	Habilidad	Objetivo	Registro etnográfico
1	Conocimiento de sí mismo	Identificar la importancia de reconocerse a sí mismos e identificar los aspectos que nos hacen únicos.	Se observó que algunas mujeres no quisieron interactuar con todos sus compañeros, y en general la dinámica se dio por grupos. La mayoría identificó la importancia de conocerse a sí mismos, y fueron capaces de mencionar al menos una cosa para la que se consideran buenos. Sin embargo, otros y otras, tuvieron algunos problemas para hacerlo. Se observó una diferencia entre hombres y mujeres, al revisar si el significado de sus nombres coincidía con sus cualidades, la mayoría de las mujeres respondía con un tono de voz bajo que "no sabían" o que "poco"" mientras que los hombres respondían con seguridad "si" o "no" según lo que consideraban. La mayoría consideró que lo más difícil fue identificar sus debilidades y fortalezas.
2		Identificar fortalezas, debilidades, gustos y disgustos propios.	
3		Aumentar el autoconocimiento.	Los estudiantes fueron capaces de describirse mejor, al grado que la mayoría pudo identificar de quién era el perfil que habían elaborado en esa sesión.
4	Empatía	Identificar qué es la empatía y la importancia de conocer a los demás.	Después de una de las actividades, comentaron que de haber sabido por todo lo que una persona había pasado "habrían cambiado su actitud" y "tratado de ser más amables". Al final de la sesión fueron capaces de conceptualizar la empatía y su importancia.
5		Aprender a conocer a los demás.	Los participantes comentaron que no tenían mucha confianza unos con otros. Se observaron diferencias en la dinámica, los hombres se mostraron menos dispuestos a platicar de cosas personales, mientras que las mujeres, refirieron que a ellas nos les costó tanto porque generalmente siempre se cuentan y platican cosas más personales entre ellas.
6		Practicar empatía.	Se hizo un reconocimiento que ellos mismo entregaron al compañero que consideraban que había sido más empático. Se reflexionó si quienes habían sido reconocidos sabían que estaban siendo empáticos. Discutieron sobre algunas situaciones difíciles y cómo se sentirían si estuvieran en esa situación.
7	Manejo de emociones	Identificar qué son las emociones. Reconocer qué emociones han experimentado.	Los y las estudiantes fueron capaces de reconocer las diferentes emociones y coincidieron en que algunas fueron más difíciles de reconocer que otras, y que una misma emoción puede expresarse de distinta manera.
8		Identificar cómo expresan sus emociones.	Reflexionaron sobre cómo hombres y mujeres expresan sus emociones. Durante esa sesión, uno de los participantes más inquietos del salón se acercó por iniciativa propia y se ofreció a ayudar a repartir los materiales.
9		Expresar las emociones de manera adecuada.	Representaron cómo una emoción se podía expresar de forma negativa y de forma positiva. Para la emoción de tristeza el equipo respondió: "No dañar ni dañarme, sería mejor escribirlo en una hoja, así podemos hacer para no ser agresivos". Para la emoción de furia, un equipo respondió: "Ignorándolos, alejándose" "Dejarlo solito cuando está enojado, así te evitas problemas" "Olvídatelo y deja ir esa furia". Se observó también que, algunos alumnos que al principio de las sesiones casi no participaban, ahora si lo hacían y expresan más sus ideas.
10	Comunicación efectiva	Identificar la importancia de la comunicación. Reconocer las formas en las que nos comunicamos.	Antes de comenzar la sesión, algunos de los estudiantes se ofrecieron a apoyar en el acomodo de las sillas, y se pusieron a platicar sobre cómo les iba en las clases. Al concluir la sesión, los estudiantes identificaron los tipos de comunicación que utilizamos y su importancia.
11		Identificar las formas de comunicación asertiva, pasiva y agresiva.	Mediante lluvia de ideas y con apoyo de la facilitadora, se expusieron los tres tipos de respuestas: asertivas, pasivas, y agresivas. Durante esta actividad, a la mayoría le dio pena realizar la dramatización. Se reflexionó sobre la mejor manera de responder.
12		Reflexionar sobre la responsabilidad que tenemos cuando nos comunicamos.	Antes de comenzar la sesión, varios alumnos se acercaron para platicar con los facilitadores sobre cuestiones personales. En la sesión escribieron varios mensajes para sus compañeros, y no se limitaron sólo a escribir mensajes a sus amigos con los que siempre están, casi todo el salón se paró a darles mensajes a compañeros y compañeras con los que casi no hablaban o convivían durante las primeras sesiones. "Me gustó mucho [la actividad] porque además d comunicarnos con nuestros compañeros, fuimos empáticos y por eso pudimos escribirles cosas buenas de ellos"(hombre).

Esto es complementario con los puntajes obtenidos en esta habilidad, en la que se logró un aumento estadísticamente significativo (Tabla 1). Al desagregarlas por sexo, este aumento fue mayor en las mujeres que en los hombres. En el GF post, el diálogo estuvo dominado por los hombres, mientras que las mujeres mostraron una actitud más tímida (registro etnográfico).

**Tabla 1:** Mediciones pre y postest de habilidades para la vida con estudiantes. 2018

Dimensión	General n=26			Hombres n=12			Mujeres n=14		
	Pretest	Postest	p- valor	Pretest	Postest	p-valor	Pretest	Postest	p-valor
	P50 (P25-P75)	P50 (P25-P75)		P50 (P25-P75)	P50 (P25-P75)		P50 (P25-P75)	P50(P25-P75)	
Conocimiento de sí mismo	4 (2-5)	8 (6-8)	<0,01	4 (2-5)	6,5 (5-8)	<0,01	4(2-5)	8 (7-8)	<0,01
Empatía	1 (0-2)	1 (0-2)	0,46	1 (0-1)	1 (0-2)	0,80	1 (0-2)	1 (1-2,5)	0,26
Manejo de emociones	9 (7-10)	9 (9-10)	0,20	9 (8-10)	9 (8-10)	0,59	9 (7-11)	9 (9-11,5)	0,05
Comunicación efectiva	6 (5-8)	7 (5-10)	0,90	6 (5-7)	5 (4-8)	0,53	7 (6-8)	7 (6-10)	0,33
Puntaje global	20 (17-23)	25 (21-30)	<0,01	19 (16-22)	21 (19-30,5)	0,16	20 (19-24)	27,5 (23-30)	<0,01

p-valor <0.10 Wilcoxon test.

### Empatia

No se observaron diferencias entre el pre y el pos test para hombres y mujeres. Los participantes del GF reportaron que se habían dado cuenta de que algunas de las actitudes que ya tenían previamente eran empáticas y consideraban que les habían ayudado a relacionarse mejor. En la dinámica del grupo se observó que, en general, las y los estudiantes al final de las sesiones mostraban interés y comprensión entre ellos (registro etnográfico).

### Manejo de emociones

Hubo un incremento estadísticamente significativo de las puntuaciones postest, en el caso de las mujeres. En el GF post, todos los participantes concordaron en que podían manejar mejor sus emociones; principalmente en el manejo de la furia, que fue la emoción que habían identificado en el GF pre como la que presentaba mayor dificultad para controlar.

### Comunicación efectiva

Después de las sesiones, todos los participantes del GF coincidieron en que cambió la forma en que se comunicaban con los demás y consideraban que les habían ayudado a establecer una mejor comunicación con las personas y su familia, aunque seguían teniendo dificultad para comunicarse con la figura paterna.

### Habilidades para la vida en conjunto

En el GF post, los participantes señalaron percibir un cambio en todas las habilidades (Cuadro 3), mientras que en el componente cuantitativo solo se encontró un incremento estadísticamente significativo en los puntajes de la HpV de *conocimiento de sí mismo* y en el puntaje global. Al hacer la comparación por sexo, las mujeres mostraron un incremento estadísticamente significativo en las puntuaciones en el postest en las habilidades de *conocimiento de sí mismo, manejo de emociones* y el puntaje global, mientras que los hombres solo lo hicieron en *conocimiento de sí mismo* (Tabla 1).

**Cuadro 3:** testimonios de habilidades para la vida de estudiantes

Habilidad	Testimonios
Conocimiento de sí mismo	“*Yo, por ejemplo, ya sé que soy muy enojón. Ya sé que hay personas que todavía no maduran totalmente y pues ya sé que me puedo enojar más fácilmente*” (Hombre, GF post).
Empatía	“*Si [me ayudó] con mi familia... me puse a ver cómo se sentían*” (Mujer, GF post). “*[apliqué la empatía] con un tío mío que nadie sabía por qué estaba triste y como que soy el que le tiene más confianza, ya después me puse en sus zapatos de él y comprendí (…)*“ (Hombre, GF post).
Manejo de emociones	“*Ya no hago las cosas que antes hacía [como por ejemplo] meterme a mi cuarto y pegarle a las paredes*” (Hombre, GF post). “*Yo antes cuando me enojaba, me enojaba con todos, y ahora me desquito llorando*” (Mujer, GF post). “*Cuando me enojo pues ya no lo demuestro como antes que me metía a mi cuarto y empezaba a hacer cualquier cosa, aventar cosas y así*” (Hombre, GF post).
Comunicación efectiva	“*Pues si no me gusta algo, se los digo a esa persona [antes no lo hacía]*” (Mujer, GF post). “*También soy más expresivo, ya les cuento... Ya como que gane la confianza*” (Hombre, GF post).
Habilidades para la vida	“*Pues aparte de la convivencia y la empatía, me llevo mejor con mis papás*” (Mujer, GF post). “*Aprendí a manejar mis emociones, a respetar a los demás compañeros, a no juzgarlos al primer vistazo, necesito conocerlos y aprender cómo son, Y aprendí también que soy empático (…) ya hay más comunicación entre las familias y nosotros, y también entre el mismo grupo*” (Hombre, GF post). “*Aprendí a comunicarme con mis compañeros y ya cuando me enojo ya no es tanto como antes, ya no me desquito con los demás*” (Mujer, GF post). “*Sí [vimos un cambio] porque los grupos ya se van juntando, porque ya no se llevan tan mal. Todo el grupo se está formando en un solo grupo*” (Hombre, GF Post). “*Ya nos hablamos más entre todos*” (Hombre, GF Post).

## DISCUSIÓN

La prevalencia de intento suicida que se encontró en nuestro estudio es más alta que la reportada a nivel internacional (6 % (IC 95 % 4,7-7,7 %) 2, así como la reportada a nivel nacional por las Ensanut 2006 (1,1%), 2012 (2,7%) y 2018 (3,9%) 3.

Con respecto al fortalecimiento de las HpV en el ámbito escolar, los resultados de este estudio son consistentes con otros realizados en poblaciones similares. Moreira y Murillo desarrollaron un programa educativo de HpV con niños y niñas en riesgo social de una casa hogar, y encontraron que esta población emplea algunas habilidades más que otras. Entre las habilidades que mayor dificultad tuvieron se encuentran el conocimiento de sí mismo y el pensamiento crítico 18. Este resultado es divergente con nuestro estudio, en el cual el conocimiento de sí mismo mostró ser una de las habilidades con mejores puntuaciones. Choque-Larrauri *et al.* reportan un aumento significativo en la asertividad y la comunicación en estudiantes adolescentes, lo cual es semejante a los resultados obtenidos en nuestra investigación 19. Corrales *et al.* realizaron una iniciativa para fortalecer las habilidades de empatía y comunicación asertiva en adolescentes de secundaria, en quienes reportan un cambio positivo en la empatía 17, lo cual es semejante con nuestros resultados.

Nuestro estudio se enfocó en fortalecer determinadas HpV en estudiantes de secundaria para hacer frente a situaciones relacionadas con la CS, lo cual es congruente con otras investigaciones que han abordado la metodologia de HpV para situaciones específicas como embarazo adolescente, distrés psicológico, adicciones, *bullying* y suicidio 20-26. Otro aspecto por considerar es la forma en la que estos estudios fueron evaluados y el objetivo de la medición, ya que algunos utilizaron un enfoque mixto 14,18, y otros fueron evaluados mediante la aplicación de cuestionarios 16-20,23-26. Los estudios que tenían por objetivo fortalecer las HpV midieron variables relacionadas con las habilidades 16,18,19, mientras que los estudios que buscaban abordar una problemática específica utilizaron instrumentos para evaluar dicha problemática 24-26. Otros estudios midieron tanto la problemática que estaban tratando como las HpV 17,20,23.

El número de sesiones educativas que se reporta en los estudios varía entre 7 y 24 13,18. En este estudio se llevaron a cabo 12 sesiones, y el principal cambio se presentó en la habilidad de conocimiento de sí mismo, y en las mujeres además en el manejo de las emociones; las otras dos habilidades no mostraron cambios estadísticamente significativos. Esto pudiera sugerir una jerarquización de las habilidades, a partir de la habilidad básica de conocerse a sí mismo, avanzando hacia el manejo de emociones, empatía y finalmente comunicación efectiva; es decir, las dos primeras pertenecen a la categoría de habilidades personales, mientras que las dos últimas hacen parte de las habilidades interpersonales, lo que permite a la persona relacionarse mejor en primera instancia consigo misma, luego con los demás y finalmente con su entorno 29,30. Estos resultados pudieran reflejar la necesidad de un programa con mayor duración, ya que la adquisición de habilidades es un proceso que requiere más tiempo desde que la persona reconoce la habilidad hasta que profundiza en la comprensión y en cómo llevarlo a la práctica. Tal como sugieren Morales y Benítez, las HpV involucran procesos cognitivos relacionados con los pensamientos y las percepciones, y estas habilidades se manifiestan de forma gradual, por lo que se necesita un periodo más largo para poder observar cambios de manera significativa 16.

En los resultados se encontró un cambio mayor en las mujeres que en los hombres, lo cual es similar a lo reportado en otros estudios 12,16,20. En contraste, Choque-Larrauri et. al no reportan diferencias entre hombres y mujeres 19. Asimismo, se observó una diferencia estadísticamente significativa solo en las mujeres en el manejo de las emociones; en este sentido, los estudios no son concluyentes sobre si existe una diferencia por sexo, sin embargo, se reconoce que esta habilidad se ve influida por procesos relacionados con la identidad de género 31,32.

De los estudios cuyo objetivo fue fortalecer las HpV, solo dos trabajaron con los estudiantes y los docentes 13,14, lo cual es diferente de nuestra investigación, que solo incluyó a los estudiantes.

Los constructos de aprendizaje vicario y autoeficacia de la TSC fueron la base del diseño de las sesiones educativas, de tal manera que con las dinámicas realizadas en las sesiones se logró que los estudiantes se sintieran capaces de realizar las habilidades trabajadas 6. Otro de los aspectos observados durante la implementación fue el papel activo de los estudiantes en todo el proceso educativo, en el cual ellos exponían lo que sabían del tema y reconstruían su conocimiento a partir de experiencias propias y de la interacción con los pares, lo cual es congruente con los fundamentos teóricos del constructivismo 7.

Nuestro estudio inició con la medición de la conducta suicida en adolescentes. Estos resultados fueron el sustento para la iniciativa educativa con estudiantes.

Si bien el cambio en las HpV no fue estadísticamente significativo para todas las habilidades, con la triangulación de los componentes cuantitativos y cualitativos se muestra que se logró un cambio positivo, aunque en distinta medida en cada una de HpV abordadas, lo cual evidencia que es posible fortalecer estas habilidades en el ámbito escolar.

La participación de los adolescentes en iniciativas educativa tales como las de HpV es necesaria para fortalecer a dicho grupo de población ante factores de riesgo de conducta suicida y brindar herramientas de apoyo. Para países como México es importante aumentar las experiencias de iniciativas en el ámbito escolar a fin de prevenir la conducta suicida en adolescentes.

### Limitaciones

Estos resultados se deben considerar a la luz de las limitaciones propias del diseño de este estudio, en el que la escuela y el grupo se seleccionaron por conveniencia; no se contó con un grupo control y no se puede aseverar que los cambios se atribuyen única y exclusivamente a los efectos de la iniciativa ♠
